# Thrombomodulin in disseminated intravascular coagulation and other critical conditions—a multi-faceted anticoagulant protein with therapeutic potential

**DOI:** 10.1186/s13054-019-2552-0

**Published:** 2019-08-15

**Authors:** Takashi Ito, Jecko Thachil, Hidesaku Asakura, Jerrold H. Levy, Toshiaki Iba

**Affiliations:** 1 Department of Systems Biology in Thromboregulation, Kagoshima University Graduate School of Medical and Dental Sciences, 8-35-1 Sakuragaoka, Kagoshima, 890-8544 Japan; 2 Department of Haematology, Manchester University Hospitals NHS Foundation Trust, Manchester, UK; 3 Third Department of Internal Medicine, Kanazawa University Graduate School of Medical Science, Kanazawa, Japan; 4 Department of Anesthesiology, Critical Care and Surgery, Duke University School of Medicine, Durham, NC USA; 5 Department of Emergency and Disaster Medicine, Juntendo University Graduate School of Medicine, Tokyo, Japan

**Keywords:** Recombinant thrombomodulin, Disseminated intravascular coagulation (DIC), Coagulopathy, Randomized controlled trial, Bleeding, Sepsis, Septic shock

## Abstract

Thrombomodulin plays a vital role in maintaining intravascular patency due to its anticoagulant, antiinflammatory, and cytoprotective properties. However, under pathological conditions such as sepsis and systemic inflammation, endothelial thrombomodulin expression is downregulated and its function impaired. As a result, administering thrombomodulin represents a potential therapeutic modality. Recently, the effect of recombinant thrombomodulin administration in sepsis-induced coagulopathy was evaluated in a randomized controlled study (SCARLET). A 2.6% 28-day absolute mortality reduction (26.8% vs. 29.4%) was reported in 800 patients studied that was not statistically significant; however, a post hoc analysis revealed a 5.4% absolute mortality reduction among the patients who fulfilled the entry criterion at baseline. The risk of bleeding did not increase compared to placebo control. Favorable effects of thrombomodulin administration have been reported not only in sepsis-induced coagulopathy but also in disseminated intravascular coagulations with various backgrounds. Interestingly, beneficial effects of recombinant thrombomodulin in respiratory, renal, and cardiovascular diseases might depend on its anti-inflammatory mechanisms. In this review, we summarize the accumulated knowledge of endogenous as well as recombinant thrombomodulin from basic to clinical aspects and suggest future directions for this novel therapeutic agent.

## Background

Ever since Esmon et al. first reported the importance of thrombomodulin (TM) as a cofactor for thrombin-catalyzed activation of protein C in the 1980s [1], this important molecule has attracted considerable interest in the field of thrombosis and inflammation. TM was initially considered an important cofactor for the anticoagulant protein C system, but subsequently, TM was noted to be a critical component of a multimolecular system, integrating major endothelial function that includes antithrombotic, antiinflammatory, and cytoprotective properties [2]. These findings made TM an attractive drug candidate for therapy of diseases where vascular endothelial function was impaired. Accordingly, a recombinant TM (rTM, ART-123, Recomodulin®) was developed and studied for the treatment of disseminated intravascular coagulation (DIC) [3]. Recently, a randomized controlled trial (RCT) that examined the effect of rTM on sepsis-induced coagulopathy was performed [4]. The results of this study and a meta-analysis reported a trend toward favorable outcomes in the treatment groups [5]. In this review, we summarize the physiological role of TM, the potential utility of soluble fragments of TM as a biomarker, and the efficacy of rTM in clinical use.

## Physiology of thrombomodulin (TM)

TM is a type I transmembrane glycoprotein composed of 557 amino acids that is expressed on the luminal surface of endothelial cells where it suppresses thrombus formation by modulating thrombin’s procoagulant effects. TM binds reversibly to the anion-binding exosite-I of thrombin, interrupting the binding of thrombin to its procoagulant substrates that include fibrinogen, protease-activated receptors, and coagulation factors V and VIII [6]. Conversely, TM provides the binding surface for protein C in such a manner that this anticoagulant zymogen is efficiently activated by thrombin bound to the adjacent portion of TM [6]. As a result, activated protein C (APC), in association with protein S, degrades coagulation factors Va and VIIIa further inhibiting thrombin generation. Endothelial-specific loss of TM results in spontaneous and fatal thrombosis in mice, suggesting that the TM-protein C pathway is critical in preventing intravascular coagulation [7].

**Fig. 1:**
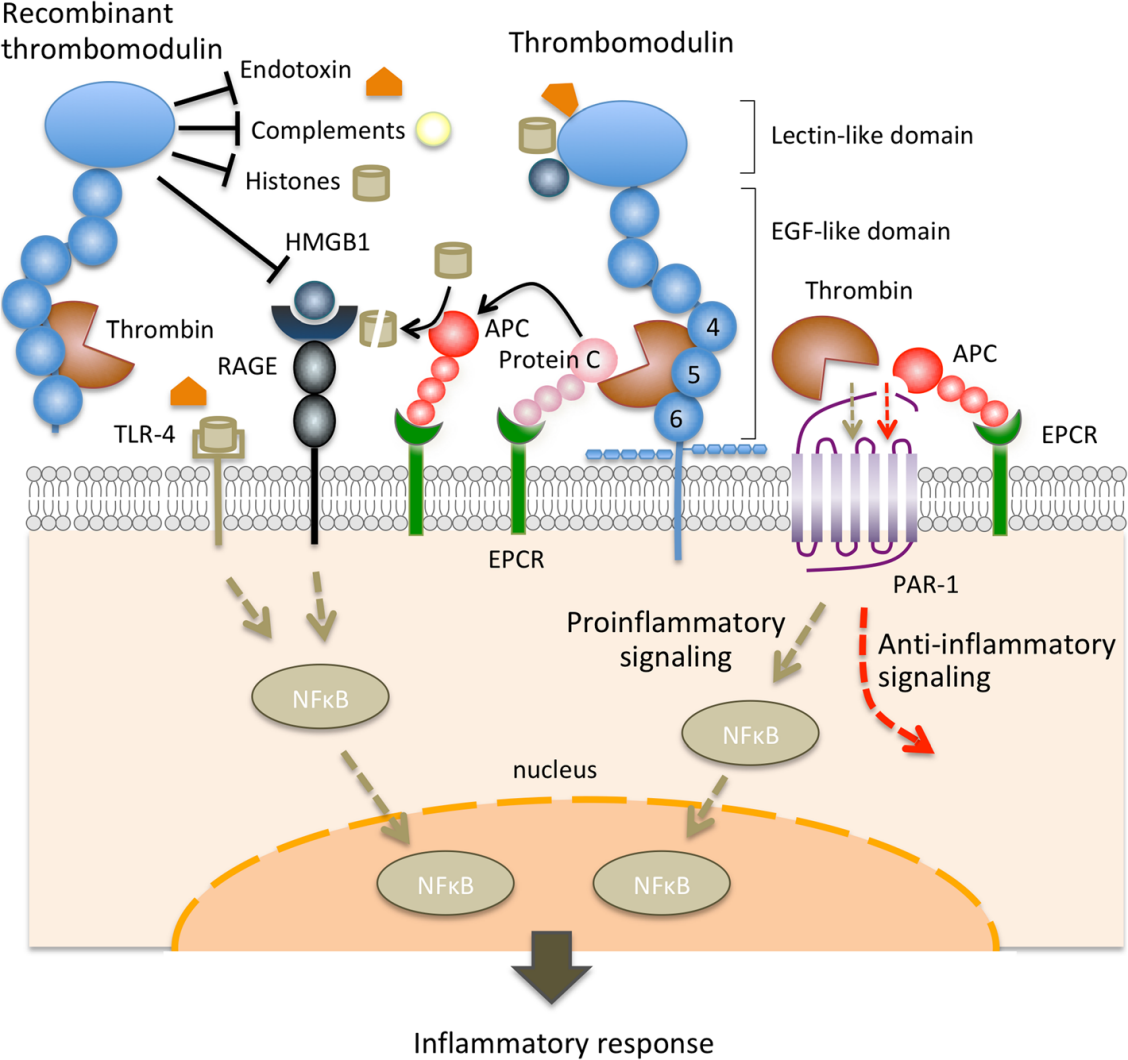
Anti-inflammatory effects of thrombomodulin. The thrombin–thrombomodulin complex activates protein C on the surface of endothelial cells, and this activation is facilitated by endothelial protein C receptor (EPCR). Although thrombin initiates proinflammatory signaling by cleaving protease-activated receptor 1 (PAR1) (brown dotted arrow), activated protein C (APC) associated with EPCR cleaves PAR1 differently and initiates cell signaling that provides anti-inflammatory effects (red dotted arrow). The lectin-like domain of TM binds high mobility group box 1 (HMGB1) and inhibits its signaling via the receptor for advanced glycation end product (RAGE). The activation of RAGE by HMGB1 initiates the nuclear translocation of nuclear factor kappa B (NF-κB) that induces an inflammatory response. Similarly, the lectin-like domain of thrombomodulin blocks the interaction between Toll-like receptor 4 (TLR-4) and its ligands, such as endotoxin and histones, thereby inhibiting proinflammatory reactions

Besides the anticoagulant effects, APC has cytoprotective and antiinflammatory effects (Fig. 1) that are mediated by the endothelial protein C receptor (EPCR)-protease-activated receptor 1 (PAR1) system [8]. PAR1 was initially identified as a receptor for thrombin, and thrombin binding induces proinflammatory responses, contributing to the pathogenesis of vascular inflammation. However, PAR1 can be alternatively activated by EPCR-APC, eliciting protective signaling responses in endothelial cells [8, 9] that include downregulation of vascular adhesion molecules, stabilization of endothelial barrier function, and inhibition of apoptotic signaling.

Thrombin activatable fibrinolysis inhibitor (TAFI) is another critical substrate for the thrombin–TM complex. Activated TAFI inhibits fibrinolysis by removing lysine residues from the carboxy-terminal region of fibrin, thereby diminishing plasminogen binding to fibrin. TAFI is also important in regulating complement activation [10]. In addition to these actions, TM has a direct inhibitory effect on inflammatory responses that is conferred by the lectin-like domain of TM. Mice lacking this domain are healthy but are vulnerable to neutrophil-mediated tissue damage with inflammatory diseases [11]. There also appears to be multiple mechanisms by which TM’s lectin-like domain suppresses inflammatory responses that include suppression of leukocyte adhesion to endothelial cells [11], interference of complement activation [12, 13], inactivation of proinflammatory nuclear proteins, such as high mobility group box 1 (HMGB1) and histones [14], and inactivation of bacterial endotoxin [15]. Interestingly, endotoxin, HMGB1, and histones are recognized by the common pattern recognition receptors, and thus TM binding inhibits the receptor-mediated proinflammatory reactions. Taken together, these findings suggest that the anti-inflammatory effects of TM arise through multi-factorial mechanisms involving both APC-dependent and APC-independent actions [14].

## Endogenous soluble TM as a potential biomarker of endothelial injury

Fragments of the extracellular region of membrane-bound TM called endogenous soluble TM can be generated by proteolytic cleavage by leukocyte-derived proteases and metalloproteases that are released in sepsis and inflammatory states [2, 16]. Although these shed fragments can be detected in plasma and urine of healthy individuals, their levels are increased several-fold under certain inflammatory conditions including trauma, DIC, pulmonary thromboembolism, acute respiratory distress syndrome (ARDS), chronic renal failure, or acute hepatic failure [17, 18]. Soluble TM may independently have some physiological functions as 30–50% of the anticoagulant activity of cell-associated TM may be preserved in these soluble fragments depending on the fragment length [19, 20].

## The development of rTM

The cDNA and amino acid sequence of human TM was first identified by Suzuki et al. in 1987 [21]. The recombinant form (rTM, ART-123, Recomodulin®), which consists of the extracellular portion of TM and lacks the hydrophobic transmembrane and cytoplasmic regions, was subsequently synthesized in Chinese Hamster Ovary cells and was evaluated as a potential anticoagulant. Compared to natural TM, rTM had an approximately equivalent binding affinity for thrombin and cofactor activity for protein C activation [21]. When dosed clinically, rTM preferentially inhibits thrombin generation through protein C activation and subsequent factor Va inactivation, without inhibiting thrombin activity directly [22]. As a result, rTM had minimal effects on clotting times compared to the direct thrombin inhibitor argatroban, indirect thrombin inhibitor heparin, or recombinant APC [22, 23]. Although rTM and APC both molecules share the same anticoagulant mechanism, rTM provides APC-dependent anticoagulation only after thrombin is generated. Since plasma APC can be rapidly inactivated by several plasma serine protease inhibitors, rTM is considered to act locally where thrombin is generated [24], a potential reason for the observed low risk of bleeding with rTM therapy compared to APC [25]. Based on the pharmacokinetic data obtained from healthy volunteers and DIC patients, the intravenous infusion of 0.06 mg/kg rTM once daily was recommended [26, 27]. The half-life of rTM is approximately 20 h, and more than 50% of the drug is excreted from urine. However, the dose reduction is not necessary even in moderate renal dysfunction [28]. rTM is currently available only in Japan and the cost for 6-day-treatment will be over $3000 in US dollars.

## The clinical effectiveness of rTM in sepsis

The theoretical background of the anticoagulant therapy for sepsis-induced coagulation dysfunction has been described in other reviews [10, 29, 30]. One of the pivotal trials in the management of septic coagulopathy was the recombinant APC (drotrecogin alfa [activated], Xigris®) study [31]. However, the most recent analysis of its efficacy and safety for use in septic patients led to its removal from clinical use [32]. The failure of the most recent recombinant APC study was due to multiple considerations including bleeding side effects, dosing, timing, and efficacy. Among all, the most important consideration is the patient selection. Anticoagulants such as recombinant APC should be effective only to the septic patients with coagulopathy or DIC [25]. Hence, subgroup analyses in large-scale RCTs have been performed to examine the effect of anticoagulants, including antithrombin and recombinant APC, in patients with sepsis and have reported trends toward a greater risk reduction in mortality among patients with DIC than among patients without DIC [33, 34].

Based on this background, a phase III, randomized, double-blind, parallel-group trial comparing rTM and heparin in 234 patients with DIC associated with infection or hematologic malignancy was performed in Japan [3]. A sub-group analysis for the septic DIC patients revealed that the absolute difference in mortality was 11.2% (rTM 21.4% vs. heparin 31.6%), but the difference was not statistically significant (95% confidence interval [CI] − 9.1–29.4%) [35]. However, since the primary endpoint, DIC resolution rate, was significantly better in the rTM group (difference 16.2%, 95% CI 3.3–29.1), the Japanese healthcare system approved rTM as a therapeutic agent for DIC in 2008. Subsequently, a multi-national RCT was conducted to examine the effect of rTM in sepsis with suspected DIC [36]. This phase IIb trial was performed in 233 ICUs in 17 countries, and a total of 750 patients with septic coagulopathy (thrombocytopenia with prolonged prothrombin time) were enrolled. The results revealed a 3.8% reduction in the absolute risk of death (rTM group: 17.8% vs. placebo group: 21.6%, *P* = 0.273). Though this study could not demonstrate a statistically significant survival benefit, it is noteworthy that coagulation biomarker levels such as D-dimer and thrombin–antithrombin complex (TAT) levels decreased in the rTM group compared to the placebo group. Hoppensteadt et al. [37] further analyzed data from this phase IIb trial and reported that prothrombin fragment 1.2 levels decreased by 16% from the baseline until day 7 in DIC patients treated with rTM, whereas the level increased by 8% in the placebo control. The phase IIb study also reported that the efficacy was more prominent in patients with prothrombin time ratio > 1.4 and organ dysfunction.

Since its approval for the treatment of DIC in Japan, the clinical effectiveness of rTM has been extensively evaluated. Yamakawa et al. [38] collected data from 12 studies (838 patients from 3 RCTs and 571 patients from 9 observational studies) and reported that the relative risk of death was 0.81 (95% CI 0.62–1.06) in the RCTs and 0.59 (95% CI 0.45–0.77) in the observational studies. Although the meta-analysis reported a trend toward favorable outcomes, inconsistent results were also reported. For example, Tagami et al. [39] performed propensity score and instrumental variable analyses using a Japanese nationwide administrative database (matched cohort of 1140 pairs) and reported that the all-cause mortality was 37.6% in the rTM treatment group vs. 37.0% in the control group (odds ratio [OR] 1.01, 95% CI 0.93–1.10). This result demonstrated that treatment with rTM did not reduce mortality among patients with pneumonia-associated DIC. Since previous studies indicate that severe cases gain more benefit from anticoagulants, the survival benefit might have been observed if the study had targeted more critically ill patients [34, 40]. Subsequently, Yoshimura et al. [41] performed a post hoc analysis using data from a multicenter retrospective cohort study and reported that the administration of rTM was significantly associated with reduced mortality among patients with a high risk of death (APACHE II score 24–29, hazard ratio [HR] 0.281, 95% CI 0.093–0.850, *P* = 0.025). In contrast, an association was not evident among lower-risk patients (APACHE II score < 24, HR 0.814, 95% CI 0.351–1.884, *P* = 0.630). They concluded that the survival benefit was obtained only in septic DIC patients with a high risk of death. Recently, Hayakawa et al. [42] collected data from 42 intensive care units and performed a propensity analysis of 452 matched pairs. The results revealed a significant association between rTM administration and a lower in-hospital mortality (OR 0.757, 95% CI 0.574–0.999, *P* = 0.049). Regarding safety features, post-marketing surveillance of rTM among 2516 septic patients with DIC demonstrated that the frequency of critical bleeding was 2.6% [43], which did not differ from that in a phase III trial performed in Japan (rTM group 1.72%, heparin group 2.61%) [3]. Based on these results, the Japanese Society on Thrombosis and Hemostasis recommends the use of rTM for DIC [44], while Surviving Sepsis Campaign Guidelines [45] and Japanese Clinical Practice Guidelines [46] for the Management of Sepsis and Septic Shock postponed the recommendation in their 2016 version.

Following these studies, the efficacy and safety of rTM in the adult patients with sepsis and coagulation disorders have been examined in a multinational, randomized, placebo-controlled, double-blinded phase III trial named SCARLET (Sepsis Coagulopathy Asahi Recombinant LE Thrombomodulin). Accordingly, a 2.6% reduction in the absolute risk of 28-day mortality (rTM group 26.8% vs. placebo group 29.4%) was reported in a total of 800 sepsis patients (rTM group, *n* = 395; placebo group, *n* = 405) with coagulopathy (platelet count < 150 × 10^9^/L and prothrombin time ratio > 1.4) and cardiovascular and/or respiratory failure, but the difference did not reach statistical difference [4]. However, it was also reported that approximately 20% of patients did not fulfill the entry criterion at baseline after initial confirmation of eligibility and that 5.4% (95% CI, − 1.68–12.48) reduction in the absolute risk of 28-day mortality was observed in patients fulfilled the entry criterion at baseline. Following the SCARLET study, Yamakawa et al. [5] updated the meta-analysis for rTM and reported that in 1762 patients enrolled, approximately 13% reduction in the risk of mortality was observed in the rTM group, but the difference was not significant (relative risk 0.87, 95% CI 0.74–1.03, *P* = 0.10, *I*^*2*^ = 0%). It is noteworthy that all clinical trials except one showed a similar trend in favor of rTM in terms of survival and a significant improvement in coagulation parameters. The finding that the effects of rTM were more prominent in patients with coagulation disorders will also support the potential of this anticoagulant. Although TM is one of the most important physiologic anticoagulants, and the effect of externally administered rTM has long been examined through the clinical trials and practice [47], further clinical study is required to prove its efficacy. We have to keep in mind that sepsis is a complex mix of heterogeneous components and therefore, it may not be possible to find a one-fit-all therapeutic agent. Like other clinical trials for the anticoagulants, the patient selection should be the key to the success in the rTM study.

## The efficacy of rTM in hematological malignancies

In contrast to sepsis-associated DIC where organ dysfunction due to microthrombosis occurs, DIC associated with hematological malignancies exhibits bleeding symptoms. Consumptive coagulopathy and profound fibrinolysis are thought to be responsible for bleeding symptoms in this previously described “enhanced-fibrinolytic-type” DIC [48]. Thus, targeted inhibition of both coagulation and fibrinolysis is important in managing enhanced-fibrinolytic-type DIC, such as acute promyelocytic leukemia (APL)-associated DIC. Specifically, nafamostat mesylate, a serine protease inhibitor that has both antithrombotic and antifibrinolytic effects, or combined therapy of a heparinoid drug and tranexamic acid, is effective against bleeding symptoms in enhanced-fibrinolytic-type DIC [49, 50]. However, in patients with complex coagulopathic states, antifibrinolytic agents have the potential risk of thrombotic complications due to fibrinolytic shutdown [51].

A clinical trial of rTM for the treatment of DIC with underlying hematologic malignancy or infection was conducted in Japan with unfractionated heparin as a control [3]. The DIC resolution rate was 66.1% in the rTM treatment group and 49.9% in the heparin treatment group. Moreover, bleeding symptoms became significantly better in the rTM group. Bleeding-related adverse events during the 7 days of treatment were 43.1% in the rTM group and 56.5% in the heparin group. Interestingly, the superiority of rTM was shown to be greater in the hematological malignancy patients than in the infection patients in this clinical trial. Factors responsible for the lower likelihood of adverse bleeding events have been proposed. One is rTM only exhibits an anticoagulant effect in the presence of thrombin [52], and antifibrinolytic TAFI, which can be activated by the thrombin–TM complex, may contribute to the suppression of fibrinolysis-related bleeding symptoms [53].

The safety and efficacy of rTM in patients with DIC and hematological malignancy (*n* = 1032) have also been reported from post-marketing surveillance [54]. In DIC patients who had bleeding symptoms at baseline, 55% were assessed as bleeding disappeared or improved after rTM treatment. DIC-related coagulation and fibrinolytic markers were also improved even in patients whose clinical course of underlying disease was unchanged or exacerbated. This is interesting because DIC generally depends on the underlying disease in cases of hematologic malignancy.

In a retrospective analysis of acute myeloblastic leukemia (*n* = 103, of whom 47 had DIC), rTM was reported to improve both DIC and the survival outcomes (*P* = 0.016) [55]. The lectin-like domain of rTM may confer this survival benefit through inhibiting HMGB1. HMGB1 is abundantly expressed in several leukemia cells, and inhibition of HMGB1 enhances the sensitivity to chemotherapy of leukemia cells [56]. The epidermal growth factor-like domain of rTM may also confer protection against leukemia by inducing growth arrest and differentiation of leukemia cells [57]. Thus, rTM might be promising both for the control of DIC and for the direct anti-leukemic effect.

All-trans retinoic acid (ATRA) has transformed the care of patients with APL. In the present context, ATRA inhibits intravascular coagulation not only by decreasing the expression of tissue factor in APL cells but also by increasing the expression of TM. ATRA also inhibits fibrinolysis by decreasing annexin II expression [58]. However, in some cases, DIC can be exacerbated by concurrent differentiation syndrome after ATRA therapy or tumor-lysis after chemotherapy. In such cases, rTM is effective and may reduce bleeding-related death in APL patients [59], possibly by enhancing the anti-fibrinolytic and anti-leukemic effects of ATRA in APL cells [60]. Thus, the administration of rTM for AML is promising in terms of both its anti-DIC and anti-APL cell effects.

In the setting of hematopoietic stem cell transplantation, rTM is helpful for the treatment of transplant-related serious complications, such as thrombotic microangiopathy [61], veno-occlusive disease (also known as sinusoidal obstruction syndrome) [62], and graft-versus-host disease (GVHD) [63]. Although the protective mechanisms of rTM in these complications remain to be fully elucidated, anti-inflammatory, as well as anticoagulant effects of rTM, may be involved. Furthermore, APC-mediated PAR activation may help in expanding regulatory T cells and in mitigating GVHD [64]. Protective roles of TM in GVHD are further supported by a cohort study showing that single-nucleotide polymorphisms within the TM gene predict mortality in patients with GVHD [65].

## The effects of rTM in solid tumor-associated DIC

TM is known to be expressed by tumor cells [66]. TM exhibits anti-cancer properties by acting on various steps in the oncologic pathway including affecting cancer cell proliferation, blocking tumor invasion, and metastasis. The underlying mechanisms are different depending on the type of malignancy, but one of the mechanisms suggested is a suppression of epithelial-mesenchymal transition, which can make cancer cells more susceptible to chemotherapy [67]. Another suggested mechanism is through the thrombin-PAR1 pathway [68]. Interestingly, low TM expression in tumor cells is correlated with poor prognosis, and up-regulation of TM expression by atorvastatin, an HMG-CoA reductase inhibitor, diminishes the tumorigenic capability of lung cancer cells [69]. In spite of these experimental findings, few human cancer trials of rTM have been performed, and most of them have been in the context of cancer-induced DIC.

In cases of DIC associated with solid tumors, the cancer is usually in the terminal stages and is often associated with systemic metastasis. DIC treatment should be considered in such cases when control of DIC can potentially extend survival. Reports on the use of rTM for solid tumor-associated DIC are still sparse [70]. Tamura et al. [71] reported a prospective one-arm study of 101 DIC patients (lung, stomach, breast, and other cancers). The DIC resolution rate with rTM therapy was 34.0%, while DIC scores improved in 55.2% of patients and worsened in 22.9%. The incidence of hemorrhage, mainly gross hematuria, related to rTM was 12.9% until day 28. Severe hemorrhage related to rTM did not occur. In 26 patients who could not be treated with anti-cancer agents, the DIC resolution rate and the rate of improvement in DIC scores were 27% and 40%, respectively. Among them, anti-cancer therapy could be restarted in four patients in whom DIC was controlled with rTM. These results suggest the potential benefit of rTM treatment for the solid tumor-associated DIC. In prostate cancer, malignant melanoma, vascular tumors, and some other cancers, life-threatening bleeding symptoms may occur due to the enhanced-fibrinolytic-type DIC [72]. In such cases, rTM, nafamostat mesylate, or combined therapy of heparinoid drug and tranexamic acid is thought to be useful in managing DIC-related bleeding symptoms.

## The efficacy of rTM in respiratory diseases

ARDS is a deadly complication of various diseases which causes severe lung inflammation. In addition to supportive care, there have not been any therapeutic advances despite the use of extracorporeal membrane oxygenation. The identification of the crucial role of HMGB1 in this regard once again brings rTM as an interesting therapeutic prospect in the ARDS setting [73]. HMGB1 levels in the lung were elevated in ARDS mice, and rTM administration decreased the development of ARDS that correlated with an increased T-reg cell population [74]. Other researchers evaluated the possible therapeutic effect of rTM in attenuating animal models of ARDS [75, 76] and reported HMGB1 and proinflammatory cytokine levels were significantly lowered by rTM administration. These findings confirm the anti-inflammatory effect of rTM by potential inhibition of HMGB1 pathway, which may subsequently be translated to better survival outcome.

The bidirectional interaction between inflammation and coagulation can play an important role in the pathophysiology of progressive pulmonary fibrosis. Both early- and late-phase administration of rTM suppressed the fibrotic process possibly modulating the thrombin effects and also by blocking the action of HMGB1 in a mouse model of bleomycin-induced lung fibrosis [77]. Recently, the effects of rTM on acute exacerbation of idiopathic pulmonary fibrosis (IPF) have attracted more attention. In addition to the injury of type II alveolar epithelial cells and capillary endothelial cells, the activation of coagulation is revealed to deeply be involved in the pathogenesis of this critical condition [78]. Furthermore, increased levels of soluble TM, plasminogen activator inhibitor-1, fibrin/fibrinogen degradation products, d-dimer, and thrombin–antithrombin complex were reported in patients with acute exacerbation of IPF [79, 80]. Based on these observations, the effects of rTM have been examined in clinical cases. First, Kataoka et al. [81] examined the efficacy of rTM in patients with acute exacerbation of IPF in a historical cohort study. Twenty patients treated with rTM for about 6 days showed significantly improved three-month mortality compared to the control group (OR 0.219; 95%CI 0.049–0.978; *P* = 0.047). A larger study with 45 patients treated with rTM confirmed similar findings (OR 0.250; 95%CI 0.091–0.685) [82]. More recently, the result of a single-arm open-label multicenter cohort study was reported, in which 90-day survival rate of in rTM-treated group was 66.7% (26/39) and that in the historical control was 47.5% (29/61) (adjusted HR 0.453; 95%CI 0.237–0.864; *P* = 0.0163) [83]. Though the concept of treating acute exacerbation of IPF with rTM is interesting, the studies are still exploratory. High-quality evidence is needed to further support additional therapeutic applications of rTM.

## The roles of TM in renal diseases

The most common disease in relation to TM is hemolytic uremic syndrome (HUS) [84], which consists of the triad of micro-angiopathic hemolytic anemia, thrombocytopenia, and renal failure, and should be distinguished from DIC [85]. Atypical HUS, which is not triggered by Shiga-toxin, constitutes about 10% of all cases and has a poor prognosis. About half of atypical HUS cases are caused by the genetic mutations in complement regulation. Delvaeye et al. [13] reported that TM can accelerate factor I-mediated inactivation of C3b and, by activating procarboxypeptidase B, speeds up the inactivation of anaphylatoxins C3a and C5a in vitro. Consistent with these findings, 5% of atypical HUS cases are caused by mutations in the TM gene [13]. Based on these findings, rTM was evaluated in three Japanese children with HUS and mitigated thrombocytopenia, hemolysis, and renal dysfunction in the three patients and decreased complement activation in one of the patients [86]. All the patients made good recovery without any neurological sequelae, abnormal renal dysfunction, or adverse effects from rTM. In an experiment with severe and moderate HUS-model mice injected with Shiga toxin and lipopolysaccharide, it was noted that rTM administration decreased mortality [87]. Hemoglobin, platelet counts, inflammatory biomarkers, biomarkers of endothelial injury, and mesangiolysis scores were improved within 24 h after the administration of rTM. Animal studies have also demonstrated protective roles of TM in the setting of pyelonephritis [88], and protective effects of rTM in a rat model of anti-glomerular basement membrane glomerulonephritis [89].

## The roles of TM in cardiovascular diseases

TM is widely distributed throughout the vasculature. However, in some organs, its expression is restricted in a vascular-bed-specific manner. The brain is one such example, and TM is sparse or even absent in blood vessels of the putamen in young subjects [90]. In mice, endothelium-specific loss of TM results in increased fibrin deposition in the lungs and heart, but not in the brain, suggesting that molecules other than TM contribute to brain vascular patency under normal conditions. Nevertheless, exogenously administered TM reduces the infarct volume in murine stroke models, possibly through its anti-inflammatory and anticoagulant actions [91, 92]. These findings suggest the potency of rTM for the treatment of acute ischemic stroke.

TM is expressed on the luminal surface of coronary arteries under normal physiologic conditions. However, TM expression is locally downregulated in atherosclerotic lesions of coronary arteries [93]. In atherogenic mice, exogenously administered TM reduces atherosclerosis and neointima formation in a thrombin-dependent manner [94]. Thus, rTM may be useful to limit the progression of atherosclerosis, although some difficulties are inherent in long-term parenteral administration of rTM.

There is controversy regarding the significance of endogenous soluble TM levels as a biomarker of cardiovascular diseases. In healthy individuals, a high concentration of soluble TM may be associated with a low risk of coronary heart disease [95]. In contrast, a high concentration of soluble TM may predict fatal outcomes of patients who have already had a brain infarction [96]. Interpretation of soluble TM values can be difficult because a high concentration of soluble TM indicates increased expression of TM on the surface of healthy endothelial cells or increased release of soluble fragments of TM from damaged endothelial cells. The former may be associated with good outcomes because of the cytoprotective effects of this protein, whereas the latter may be associated with poor outcomes because of endothelial damage. Therefore, soluble TM values should be interpreted according to the vascular history of patients [96].

## Conclusion

The discovery of TM has facilitated a new era of research focusing on the crosstalk between coagulation and inflammation. It has also formed the basis of a therapeutic model that could be targeted to clinical states associated with endothelial dysfunction. The earliest trials have been in the classical disease state of DIC, where the link between inflammation and coagulation has been extensively studied (Table 1). The success of rTM in the DIC setting, notably with the tolerable side effects profile, divides an important therapeutic approach in this complex coagulopathy. Additional studies continue to determine the optimal role for rTM in the therapeutic armamentarium for disease conditions where endothelial dysfunction coupled with proinflammatory states exist. However, further randomized controlled trials with adequate patient numbers are required to better define the therapeutic role of rTM. On a related note, standardization of soluble TM measurements is required prior to its use as a marker of endothelial injury, because the interpretation of the soluble TM level remains controversial.

**Table 1:** Summaries of RCTs and observational studies on rTM

Summary of RCTs					
Disease	Source	Intervention		Mortality (%)	Resolution of DIC
		rTM	Control		
Sepsis	Aikawa et al. [35]	0.06 mg/kg/day for 6 days (*n* = 42)	UFH (*n* = 38)	21.4% vs 31.6%	73.2% vs 63.2%
	Vincent et al. [36]	0.06 mg/kg/day for 6 days (*n* = 370)	Placebo (*n* = 371)	17.8% vs 21.6%	28.9% vs 18.9% (day 1)
	Hagiwara et al. [97]	0.06 mg/kg/day up to 6 days (*n* = 45)	w/o rTM (*n* = 47)	17.0% vs 15.6%	*90.7% vs 65.9%
	Vincent et al. [4]	0.06 mg/kg/day for 6 days (*n* = 395)	Placebo (*n* = 405)	26.8% vs 29.4%	N/A
Hemotologic malignancy	Saito et al., [3]	0.06 mg/kg/day for 6 days (*n* = 64)	UFH (*n* = 61)	17.2% vs 18.0%	*65.6% vs 45.9%
Summary of observational studies					
Disease	Source	Intervention		Results (95% CI)	
		rTM	Control		
Sepsis	Ohryoji et al. [38]	0.06 mg/kg/day for 6 days (*n* = 17)	w/o rTM (*n* = 16) historical control	Mortality rate 23.5% vs 50.0%	
	Yada et al. [38]	0.06 mg/kg/day for 6 days (*n* = 12)	w/o rTM (*n* = 16)	30-day mortality rate 25.0% vs 18.8%	
	Kudo et al. [38]	0.06 mg/kg/day for 6 days (*n* = 30)	w/o rTM (*n* = 23) historical control	30-day mortality rate *10.0% vs 34.8%	
	Umegaki et al. [38]	0.06 mg/kg/day for 7 days (*n* = 33)	Danaparoid (*n* = 40) historical control	28-day mortality HR 0.72 (0.31–1.66)	
	Yamakawa et al. [98]	0.06 mg/kg/day for 6 days (*n* = 68)	w/o rTM (*n* = 94)	In-hospital mortality *HR 0.45 (0.26–0.77)	
	Kato et al. [99]	0.06 mg/kg/day for 7 days (*n* = 12)	w/o rTM (n = 23)	28-day mortality *HR 0.384 (0.088–0.904)	
	Sawano et al. [38]	0.06 mg/kg/day for 6 days (*n* = 51)	w/o rTM (*n* = 60)	28-day mortality *HR 0.28 (0.11–0.72)	
	Yamato et al. [100]	0.06 mg/kg/day for 6 days (*n* = 14)	w/o rTM (*n* = 8) historical control	28-day mortality rate *14.3% vs 62.5%	
	Tagami et al. [39]	0.06 mg/kg/day for 6 days (*n* = 1140)	w/o rTM (*n* = 1140)	28-day mortality OR 1.01 (0.93–1.10)	
	Hayakawa et al. [42]	0.06 mg/kg/day for 6 days (*n* = 452)	w/o rTM (*n* = 452)	In-hospital mortality *OR 0.757 (0.574–0.999)	
AML	Takezako et al. [55]	0.06 mg/kg/day for 6 days (*n* = 14)	LMWH (*n* = 33)	Estimated overall survival *Superior in rTM	
AE of IPF	Kataoka et al. [81]	0.06 mg/kg/day for 6 days (*n* = 20)	w/o rTM (*n* = 20) historical control	3-month mortality *OR 0.219 (0.049–0.978)	
	Sakamoto et al. [82]	0.06 mg/kg/day for 6 days (*n* = 45)	w/o rTM (*n* = 35) historical control	3-month mortality *OR 0.250 (0.091–0.685)	
AE of IIP	Arai et al. [83]	0.06 mg/kg/day for 6 days (*n* = 39)	w/o rTM (*n* = 61) historical control	90-day mortality *HR 0.453 (0.237–0.864)	

*DIC* disseminated intravascular coagulation, *rTM* recombinant thrombomodulin, *UFH* unfractionated heparin, *N/A* not available, *CI* confidence interval, *AML* acute myeloblastic leukemia, *AE* acute exacerbation, *IPF* idiopathic pulmonary fibrosis, *IIP* idiopathic interstitial pneumonia, *LMWH* low molecular weight heparin, *HR* hazard ratio, OR odds ratio

*Statistically significant

## Data Availability

Not applicable.

## Abbreviations

APACHE II score: Acute physiology and chronic health evaluation II score
APC: Activated protein C
APL: Acute promyelocytic leukemia
ARDS: Acute respiratory distress syndrome
ATRA: All-trans retinoic acid
CI: Confidence interval
DIC: Disseminated intravascular coagulation
EPCR: Endothelial protein C receptor
GVHD: Graft-versus-host disease
HMGB1: High mobility group box 1
HR: Hazard ratio
HUS: Hemolytic uremic syndrome
OR: Odds ratio
PAR1: Protease-activated receptor 1
RCT: Randomized controlled trial
rTM: Recombinant thrombomodulin
TAFI: Thrombin activatable fibrinolysis inhibitor
TAT: Thrombin–antithrombin complex
TM: Thrombomodulin
